# Honeymoon Period in Newborn Rats With CDH Is Associated With Changes in the VEGF Signaling Pathway

**DOI:** 10.3389/fped.2021.698217

**Published:** 2021-07-16

**Authors:** Karina Miura da Costa, Alexandre Todorovic Fabro, Christiane Becari, Rebeca Lopes Figueira, Augusto F. Schmidt, Rodrigo Ruano, Lourenço Sbragia

**Affiliations:** ^1^Laboratory of Experimental Fetal Surgery, Division of Pediatric Surgery, Department of Surgery and Anatomy, Ribeirão Preto Medical School, University of São Paulo, São Paulo, Brazil; ^2^Department of Pathology, Ribeirão Preto Medical School, University of São Paulo, São Paulo, Brazil; ^3^Division of Vascular and Endovascular Surgery, Department of Surgery and Anatomy, Ribeirão Preto Medical School, University of São Paulo, São Paulo, Brazil; ^4^Division of Neonatology, University of Miami Miller School of Medicine, Miami, FL, United States; ^5^Division of Maternal-Fetal Medicine, Department of Obstetrics and Gynecology, Mayo Clinic, Rochester, MN, United States

**Keywords:** congenital diaphragmatic hernia, eNOS enzyme, VEGF, VEGFR-1, VEGFR-2, ventilation

## Abstract

**Background:** Patients with congenital diaphragmatic hernia (CDH) have a short postnatal period of ventilatory stability called the honeymoon period, after which changes in pulmonary vascular reactivity result in pulmonary hypertension. However, the mechanisms involved are still unknown. The aim of this study was to evaluate mechanical ventilation's effect in the honeymoon period on VEGF, VEGFR-1/2 and eNOS expression on experimental CDH in rats.

**Materials and Methods:** Neonates whose mothers were not exposed to nitrofen formed the control groups (C) and neonates with left-sided defects formed the CDH groups (CDH). Both were subdivided into non-ventilated and ventilated for 30, 60, and 90 min (*n* = 7 each). The left lungs (*n* = 4) were evaluated by immunohistochemistry of the pulmonary vasculature (media wall thickness), VEGF, VEGFR-1/2 and eNOS. Western blotting (*n* = 3) was performed to quantify the expression of VEGF, VEGFR-1/2 and eNOS.

**Results:** CDH had lower biometric parameters than C. Regarding the pulmonary vasculature, C showed a reduction in media wall thickness with ventilation, while CDH presented reduction with 30 min and an increase with the progression of the ventilatory time (honeymoon period). CDH and C groups showed different patterns of VEGF, VEGFR-1/2 and eNOS expressions. The receptors and eNOS findings were significant by immunohistochemistry but not by western blotting, while VEGF was significant by western blotting but not by immunohistochemistry.

**Conclusion:** VEGF, its receptors and eNOS were altered in CDH after mechanical ventilation. These results suggest that the VEGF-NO pathway plays an important role in the honeymoon period of experimental CDH.

## Introduction

Congenital diaphragmatic hernia (CDH) has an incidence of 1.09–2.67:10,000 live births (1–3), with a survival rate of 61% (4). The degree of lung hypoplasia and pulmonary hypertension (PH) are the main determinants of neonatal outcome in CDH (5, 6). Newborns with CDH often experience a honeymoon period during which they have adequate oxygenation. The mechanisms involved in this period are not clearly understood, but they could shed light on potential therapeutic strategies to improve oxygenation and ventilation in CDH.

Vascular endothelial growth factor (VEGF) and nitric oxide (NO) play a central role in the pathogenesis of PH in CDH. VEGF regulates angiogenesis, vasculogenesis, and vascular remodeling, and acts by binding to the receptors VEGFR-1 (VEGF receptor 1, also known as fins-like tyrosine kinase—Flt-1) and VEGFR-2 (VEGF receptor 2, also called fetal liver kinase—Flk-1) (7–11). VEGFR-1 reduces cell proliferation and organizes branching and the capillary network, while VEGFR-2 is responsible for vascular proliferation, promotes vascular branching and maintains endothelial cells (12–14). VEGF can stimulate the production of endothelial NO synthase (eNOS or NOS3) and, consequently, of NO (a major regulator of smooth muscle cell proliferation) (11, 15), which, in turn, upregulates VEGF (8).

Understanding how PH begins and which mechanisms lead to vascular remodeling is key to better treatment strategies in CDH (16). Given the critical role of VEGF, its receptors and eNOS on vasculogenesis and angiogenesis, the aim of this study was to evaluate the effect of mechanical ventilation during the honeymoon period on the expression of these molecules and vascular reactivity in the nitrofen CDH model.

## Materials and Methods

### Animal Model of CDH

After approval by the Committee on Ethics in the Use of Animals of Ribeirão Preto Medical School (CEUA #088/2017), CDH was induced in Sprague Dawley rats by the administration of 100 mg of nitrofen (2,4-dichlorophenyl-p-nitrophenyl ether, Maybridge^®^, Cambridge, United Kingdom) dissolved in 1 ml of olive oil by oral gavage on gestational day (GD) 9.5.

### Experimental Groups

Rats were delivered by cesarian section on GD 21.5. Neonates were separated into eight groups with *n* = 7 neonates in each: non-ventilated controls (C); controls ventilated for 30 min (C30), 60 min (C60), or 90 min (C90), all from dams that were not exposed to nitrofen; non-ventilated neonates with CDH (CDH), and neonates with CDH ventilated for 30 min (CDH30), 60 min (CDH60) and 90 min (CDH90), all with left side defects. The presence and the side of the defect were determined by transillumination of the chest (17).

### Harvest

After the cesarean section on GD 21.5, a tracheostomy was performed for neonates in the ventilated groups. A 24 G catheter was placed, connected to MiniVent type 845 (Hugo Sachs Elektronik—Harvard Apparatus GmbH, March-Hugstetten, Germany), with a frequency of 80 breaths per minute, FiO2 1.0, inspiration-expiration ratio 1:1, and PEEP 0 cmH2O. The tidal volume was 13.5 ml/kg in the control groups (75 μl) and 9 ml/kg (50 μl) in the groups with CDH, based on a previous study by our laboratory (18). Non-ventilated animals were delivered by cesarian section, weighted, and immediately euthanized. After weighing (non-ventilated groups) or completing ventilation, the neonates were euthanized by decapitation. The lungs were removed and weighed, and the left lungs were fixed for histological, immunohistochemical (IHC) or snap-frozen for molecular processing.

### Morphometry

Body weight (BW), total lung weight (TLW), left lung weight (LLW) and the ratios TLW/BW and LLW/BW were measured.

### Immunohistochemistry

Histological sections from the left lung (*n* = 4 neonates from each group) were deparaffinized in xylol and dehydrated with ethanol. Endogen peroxidase blockage was prepared by incubating the slides in a 10% solution of hydrogen peroxidase (3%) and phosphate-buffered saline (PBS) for 10 min. Antigen retrieval was performed by the heat-mediated method: the slides were placed in 10 mM citrate buffer, pH 6.0 for 40 min in Optisteam Plus steamer (model 652, Krups North America, USA), then cooled in an ice-water bath for 15 min and washed in distilled water. After that, slides were incubated in a 10% goat serum blocking solution, diluted in phosphate-buffered saline (PBS) for 30 min to block non-specific binding sites. Depending on the protein studied, the sections were incubated with the following primary antibodies: mouse anti-VEGF 1:50 in 1% bovine serum albumin (BSA) (sc-7269, Santa Cruz Biotechnology, Santa Cruz, California, USA), rabbit anti-VEGFR-1 1:50 in 1% BSA (sc-316, Santa Cruz Biotechnology, Santa Cruz, California, USA), mouse anti-VEGFR-2 1:50 in 1% BSA (sc-6251, Santa Cruz Biotechnology, Santa Cruz, California, USA), mouse anti-SMA 1:200 (clone 1A4, Santa Cruz Biotechnology, Santa Cruz, California, USA) or rabbit anti-eNOS 1:100 in 1% BSA (sc-654, Santa Cruz Biotechnology, Santa Cruz, California, USA) at 4°C overnight. After being washed, the sections were incubated in a secondary anti-mouse antibody conjugated with horseradish peroxidase 1:100 in 1% BSA (sc-2005, Santa Cruz Biotechnology, Santa Cruz, California, USA) or anti-rabbit 1:200 in 1% BSA (sc-2004, Santa Cruz Biotechnology, Santa Cruz, California, USA) for 2 h. As a negative control, the primary antibody was omitted. Vectastain ABC (Vector Labs, Burlingame, California, USA) and DAB (Sigma-Aldrich, Saint Louis, Missouri, USA) kits were used. The slides were counterstained with Harris's hematoxylin, dehydrated and assembled. They were photographed using a Nikon Eclipse 80i photomicroscope (Nikon Instruments Inc., Melville, New York, USA) with 40× magnification, and the images were captured using NIS-Elements (Nikon Corporation, Tochigi, Japan).

### IHC Evaluation of Pulmonary Vasculature—SMA Staining

The external diameter (ED) (delimited by the external elastic lamina) and the internal diameter (ID) (delimited by the internal elastic lamina) of ten vessels per animal were measured using Fiji Image J (version 2.0.0-rc-67/1.52d, National Institutes of Health, Bethesda, Maryland, USA). The media wall thickness (MWT) was calculated using the formula: MWT = (ED–ID)/ED (19, 20).

### IHC Evaluation of VEGF, VEGFR-1/2 and eNOS Expression

IHC analysis of VEGF, VEGFR-1/2 and eNOS were performed based on stereology principles, using the 50 hits Weibel reticulum (21). For each slide studied, 10 random fields were photographed. The calculation was made by counting the number of times the reticulum lines crossed the parenchyma, marked by the IHC and the result was divided by the number of lines in the reticulum crossing the lung parenchyma (22, 23).

### Western Blotting

Left lungs from *n* = 3 animals per group were homogenized in 1 ml/organ of extraction buffer (pH 7.4) containing 100 mM Tris, 100 mM sodium pyrophosphate, 100 mM sodium fluoride, 10 mM EDTA, 10 mM sodium vanadate, and 2 mM phenylmethylsulfonyl fluoride (PMSF), 0.1 mg/ml aprotinin, and 100 mM Triton-X 1% at 4°C with a tissue homogenizer (Bio-Gen PRO200, Pro Scientific, Oxford, USA), operated at maximum speed for 30 s. The extracts were centrifuged at 12,000 rpm at 4°C (Hettich Mikro 200R, Andreas Hettich GmbH & Co.KG, Tuttlingen, Germany) for 30 min to remove insoluble material, and the supernatants of these tissues were used for protein quantification using the Bradford method. Then, 50 μg of protein were denatured, run on SDS-PAGE gel electrophoresis, and transferred to nitrocellulose membranes in two stages at 100 V for 1 h each. The membranes were blocked for 1 h in Intercept^®^ (PBS) blocking buffer (#927-70001, Li-Cor Biosciences, Lincoln, NE, USA) at room temperature. After this period, they were incubated with VEGF (1:1000, ab1316, Abcam, Burlingame, CA, USA), VEGFR-1 (1:1,000, Ptglab #13687-1-AP, Proteintech Group Inc., Rosemont, IL, USA), VEGFR-2 (1:1,000, Cell Signal #9698S, Cell Signaling Technology, Danvers, MA, USA) or eNOS (1:2,000, ab76198, Abcam, Burlingame, CA, USA), and β-Actin (1:10,000, ab8226, Abcam, Burlingame, CA, USA). All antibodies were diluted in Intercept^®^ (PBS) blocking buffer (#927-70001, Li-Cor Biosciences, Lincoln, NE, USA) and the membranes were incubated at 4°C overnight. After this period, the membranes were washed in TBST 1 × and incubated with IRDye 680RD goat anti-rabbit (1:10,000, #925-68071, Li-Cor Biosciences, Lincoln, NE, USA) or IRDye^®^ 800CW donkey anti-mouse (1:10,000, #926-32212, Li-Cor Biosciences, Lincoln, NE, USA) in Intercept^®^ (PBS) blocking buffer (#927-70001, Li-Cor Biosciences, Lincoln, NE, USA) for 1 h at room temperature, and washed with TBST 1 ×. Images were acquired with Odyssey CLX Imaging System (LI-COR Corporate, Lincoln, NE, United States) and analyzed using Image Studio Lite software (version 5.2, LI-COR Inc., Lincoln, Nevada, USA). Protein expression was normalized with a loading control (β-actin).

### Statistical Analysis

Data were analyzed using ANOVA with Tukey-Kramer post-test and expressed as mean and standard deviation (SD). A *p*-value <0.05 was considered significant. The statistical analyses were performed using GraphPad Prism version 8.4.0 (GraphPad Software Inc., La Jolla, California, USA).

## Results

Twenty-nine adult female rats were required: seven not exposed to nitrofen, which generated 72 neonates, and 22 exposed to nitrofen, which generated 195 newborns.

### Morphometry

**Figure 1:**
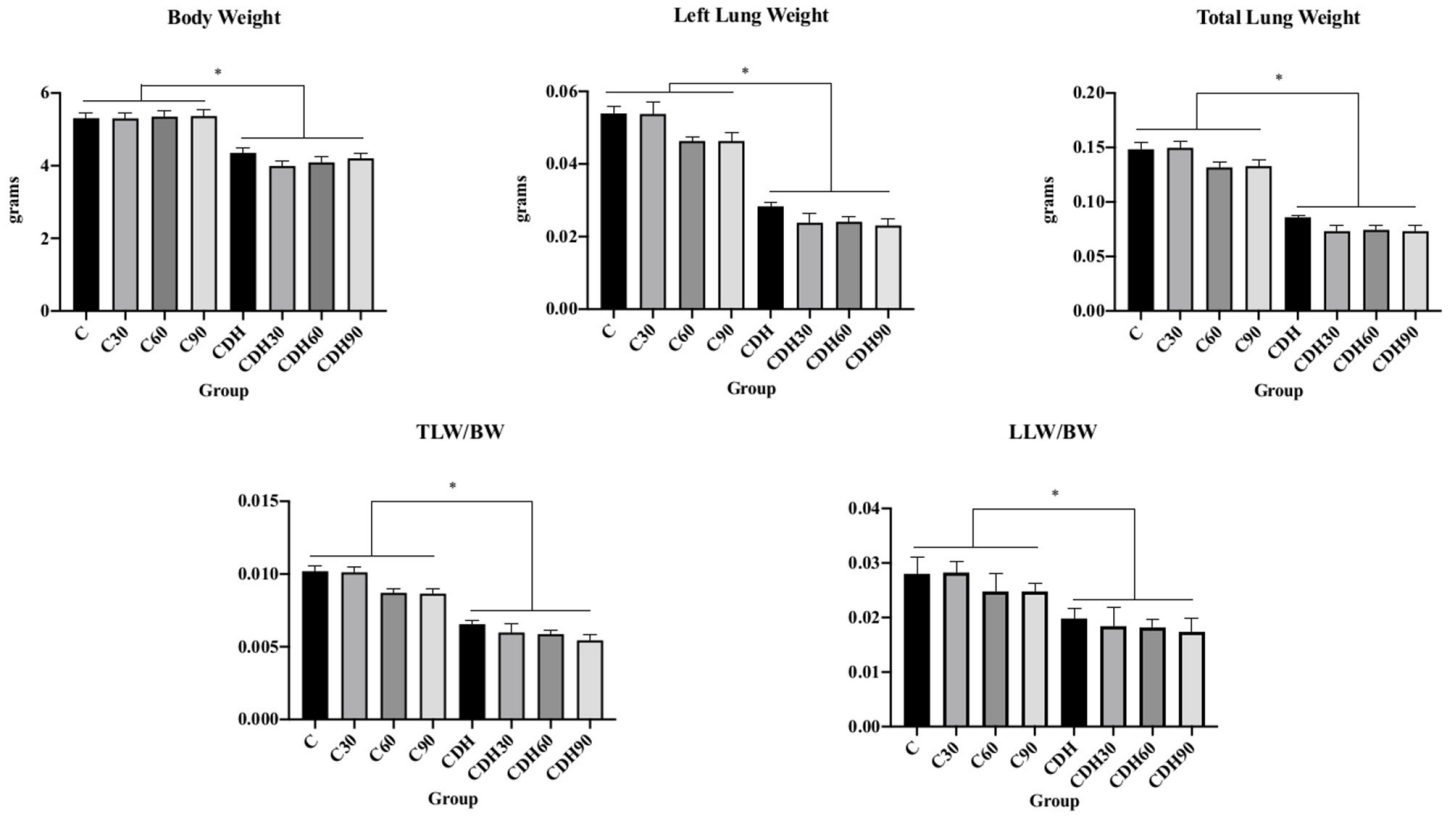
Nitrofen-induced CDH decreases birth weight and causes pulmonary hypoplasia. Newborn rats with nitrofen-induced CDH had lower body weight, left lung weight, total lung weight, total lung weight to body weight ratio (TLW/BW), and left lung weight to body weight ratio (LLW/BW). C, control; C30, control ventilated for 30 min; C60, control ventilated for 60 min; C90, control ventilated for 90 min; CDH, congenital diaphragmatic hernia; CDH30, CDH ventilated for 30 min; CDH60, CDH ventilated for 60 min; CDH90, CDH ventilated for 90 min. *p < 0.05.

Newborns with CDH had lower birth weight, lower lung weight and lower lung to body weight ratios, showing significant pulmonary hypoplasia compared to controls (Figure 1).

### MWT Measurement

**Figure 2:**
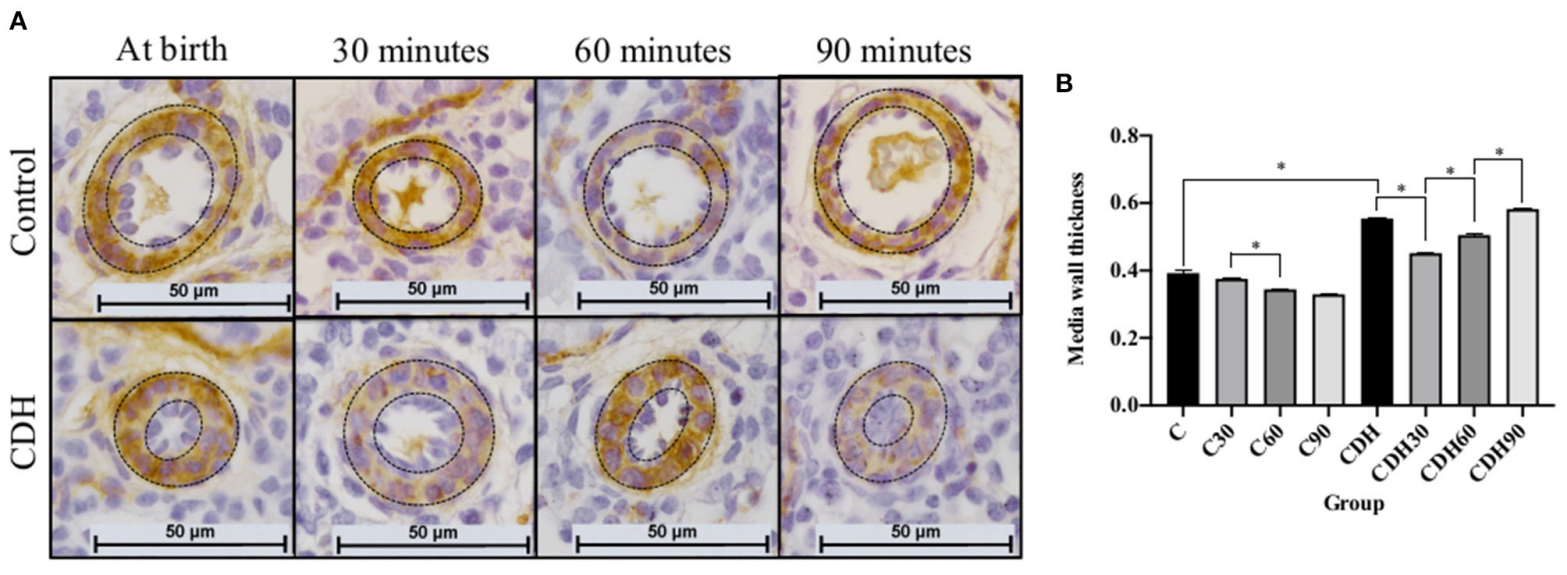
Mechanical ventilation induces a transient improvement of the media wall thickness (MWT) of the pulmonary arteries in the CDH. **(A)** Photomicrographs of representative histological sections showing pulmonary arteries immunostained for smooth muscle actin (SMA). **(B)** MWT measurement of pulmonary arteries on immunostained sections showing increased MWT in CDH animals with improvement in ventilated animals at 30 and 60 min and return to levels similar to non-ventilated CDH animals after 90 min of mechanical ventilation (*n* = 10 arteries measured in four animals per group). Dashed lines refer to the arteries' media wall thickness. CDH, congenital diaphragmatic hernia. Magnification = ×400; bar = 50 μm. C, control; C30, control ventilated for 30 mins; C60, control ventilated for 60 min; C90, control ventilated for 90 min; CDH, congenital diaphragmatic hernia; CDH30, CDH ventilated for 30 min; CDH60, CDH ventilated for 60 min; CDH90, CDH ventilated for 90 min.

MWT was measured in histological sections immunostained for SMA (Figure 2A). Non-ventilated newborn rats with CDH had increased MWT compared to non-ventilated controls (C vs. CDH, *p* < 0.001), with a decrease in the MWT at 30 min of ventilation (CDH vs. CDH30, *p* < 0.001) followed by a progressive increase at 60 min (CDH30 vs. CDH60, *p* < 0.001), and 90 min of ventilation, when it reached values similar to non-ventilated animals (CDH60 vs. CDH90, *p* < 0.001). In the controls, there was a reduction in MWT between 30 and 60 min (C30 vs. C60, *p* < 0.001), as seen in Figure 2B.

### Expression of VEGF, VEGFR-1/2 and eNOS

**Figure 3:**
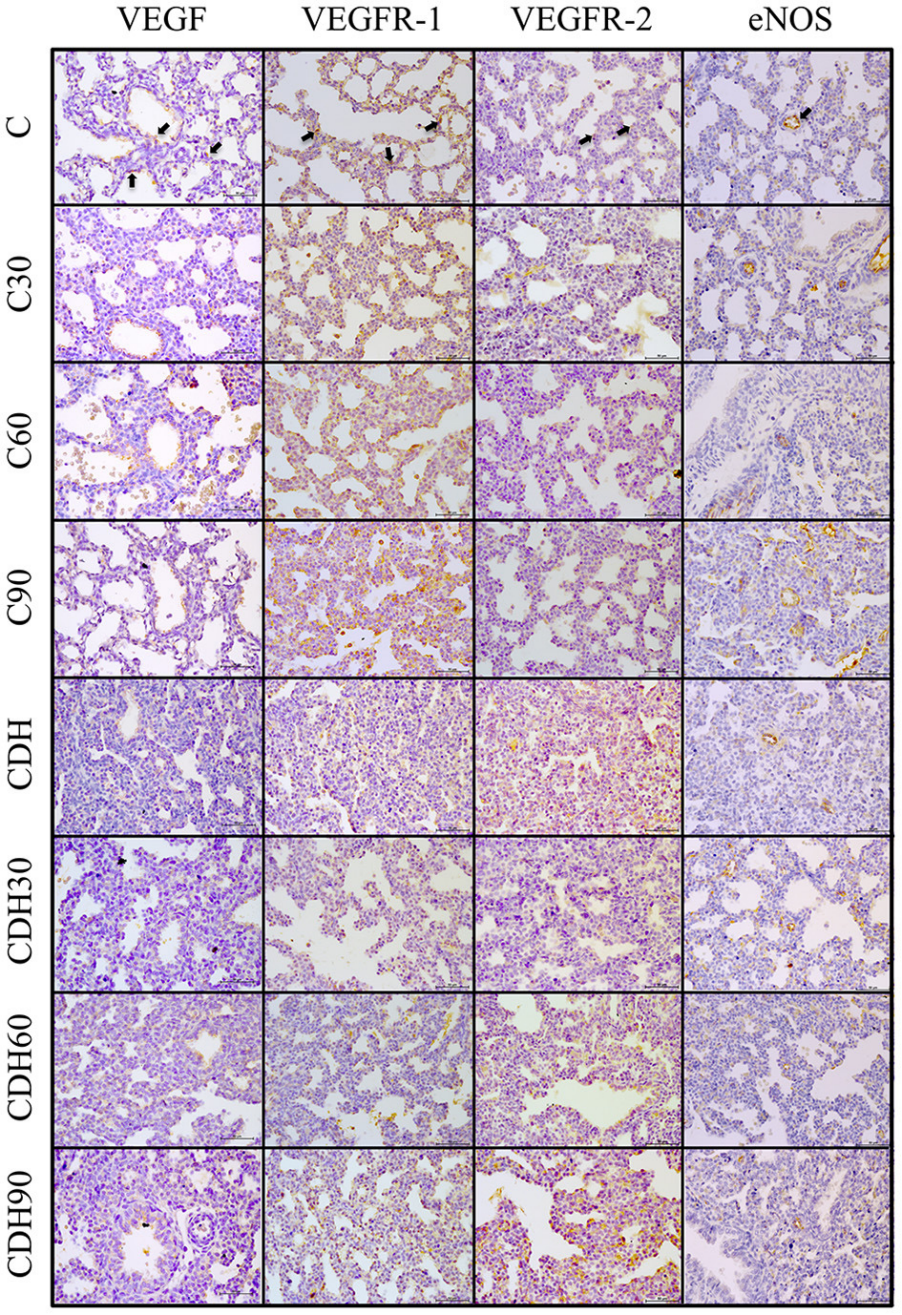
Positive immunostaining in the different groups. Representative lung sections of immunohistochemical staining for VEGF, VEGFR-1/2 and eNOS. Black arrows on the top figures show the immunostaining pattern from each marker. C, control; C30, control ventilated for 30 min; C60, control ventilated for 60 min; C90, control ventilated for 90 min; CDH, congenital diaphragmatic hernia; CDH30, CDH ventilated for 30 min; CDH60, CDH ventilated for 60 minu; CDH90, CDH ventilated for 90 min. Magnification = ×400; bar = 50 μm.

**Figure 4:**
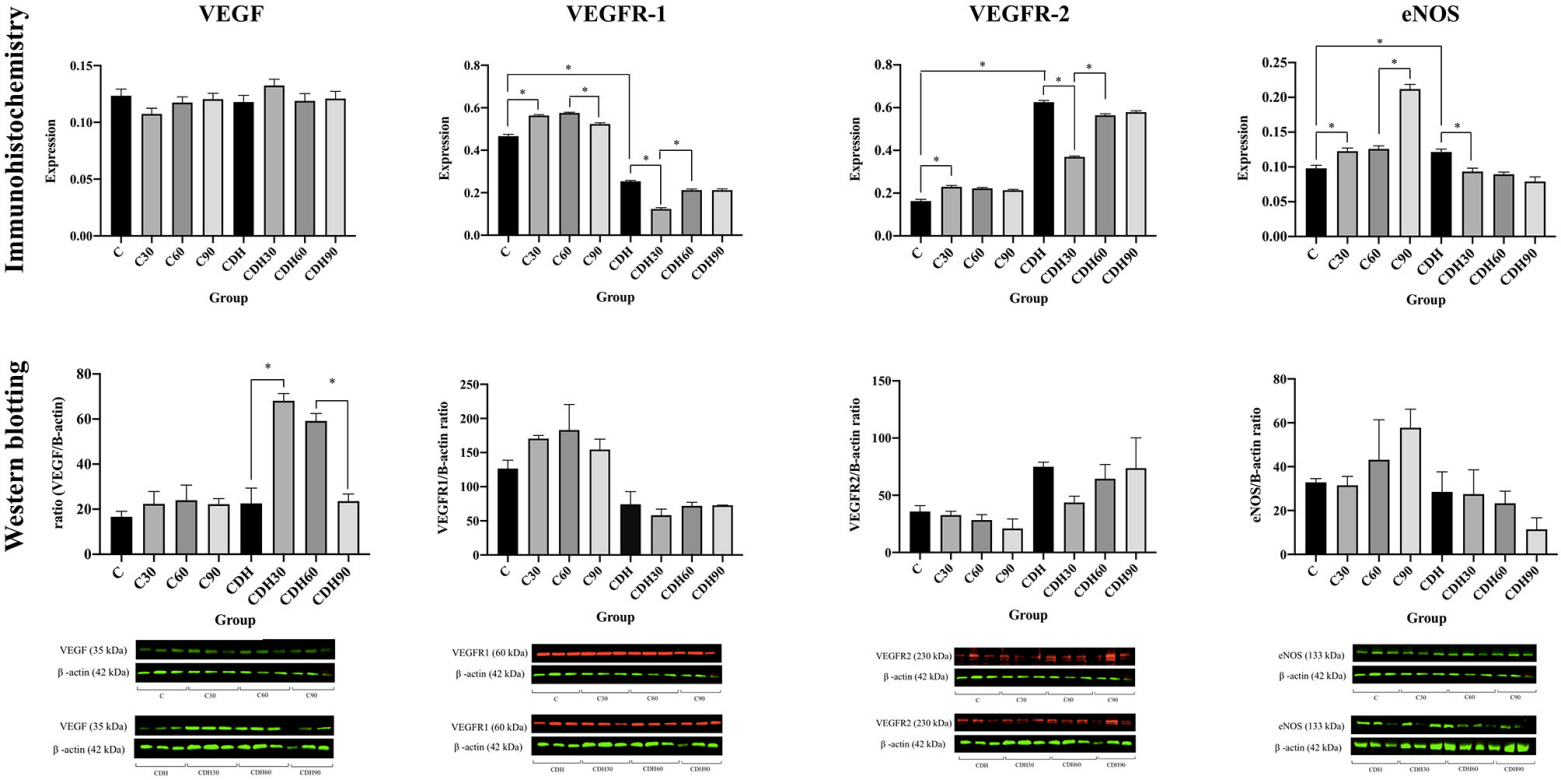
VEGF, VEGFR-1, VEGFR-2, and eNOS immunostaining and Western blotting counts. VEGF, VEGFR-1/2 and eNOS immunostaining and western blotting counts per group. C, control; C30, control ventilated for 30 min; C60, control ventilated for 60 min; C90, control ventilated for 90 min; CDH, congenital diaphragmatic hernia; CDH30, CDH ventilated for 30 min; CDH60, CDH ventilated for 60 min; CDH90, CDH ventilated for 90 min.

We observed an increased expression of VEGF at 30 min of ventilation in the groups with CDH (CDH vs. CDH30, *p* < 0.01) and a reduction after 60 min (CDH60 vs. CDH90, *p* < 0.01) in WB, but without statistical difference by IHC. There was no difference in the expression of VEGF among control animals (Figures 3, 4).

Assessment of VEGF receptors expression showed that VEGFR-1 increased with ventilation at 30 min in the controls (C vs. C30, *p* < 0.01), followed by a reduction in VEGFR-1 expression with the progression of ventilation toward 90 min (C60 vs. C90, *p* < 0.01). The groups with CDH showed a decrease of VEGFR-1 at 30 min of ventilation (CDH vs. CDH30, *p* < 0.01) with a subsequent increase (CDH30 vs. CDH60, *p* < 0.01). Concerning VEGFR-2, controls showed an increased expression of VEGFR-2 with ventilation at 30 min (C vs. C30, *p* < 0.01). In the CDH animals, there was a reduction of VEGFR-2 expression with ventilation at 30 min (CDH vs. CDH30, *p* < 0.01) and an increase after that period (CDH30 vs. CDH60, *p* < 0.01).

In the controls, there was an increase in eNOS expression with ventilation (C vs. C30, *p* = 0.01; C60 vs. C90, *p* < 0.01), while the groups with CDH presented a reduction in its expression (CDH vs. CDH30, *p* < 0.01).

Although WB expression presents a similar pattern to the IHC for VEGFR-1/2 and eNOS, there was no significant difference, possibly due to sample size and the less quantitative nature of immunohistochemistry.

## Discussion

Advances in the care of newborns with CDH have led to an increase in survival, but mortality is still high and mainly caused by PH (24). Hence, understanding the pathological changes that determine the beginning of this PH will lead to improved therapeutic strategies in newborns with CDH. We describe that the honeymoon period in fetuses with CDH is associated with changes in VEGF expression, receptors, and eNOS, suggesting a mechanism that can be explored for future therapeutic interventions.

Our morphometric results were consistent with previous publications, which observed lower birth weight and pulmonary hypoplasia in newborn rats with nitrofen induced CDH (16, 25–28).

The ventilation periods used in our study were designed to study the critical period of adaptation from fetal to neonatal circulation, which happens in the first 24 h of the life of the human newborn (29). In the newborn rat life, 30 min corresponds to about 13 h of a human newborn; 60 min to about 26 h of the human newborn; and 90 min to about 40 h of the human newborns (29, 30).

Previous studies have evaluated the MWT of newborn rats with CDH after 30 min of ventilation, with results similar to those found here (16, 26–28, 31, 32). However, the mechanisms of these changes in the pulmonary vasculature are not yet understood (33), and no study had evaluated this parameter with continued ventilation for 60 and 90 min. Controls showed a reduction in MWT (vasodilation) with ventilation. In the rat pups with CDH, despite the decreased MWT at 30 min, the response was not sustained after 60 and 90 min, and MWT increased again. These findings suggest that there is, in fact, a pathological vascular adaptation in patients with CDH, a group in which the response to ventilation corresponds to vasoconstriction, which may be one of the causes of the persistence of the fetal pulmonary circulation pattern (34). We did not find publications that described this phenomenon in rats with CDH, and the hypothesis is that it corresponds to the honeymoon period.

Little is known about the molecular mechanisms involved in the honeymoon period of patients with CDH and the changes that culminate in their end. This phenomenon was first described by Collins et al., who questioned the hypothesis that pulmonary hypoplasia was solely responsible for the CDH patients' high morbidity and mortality because, in isolation, it did not explain the transient clinical stability in the first hours of life. They postulated that this period's pathophysiology is likely due to a dynamic event, not only due to pulmonary hypoplasia (35). Due to the role of VEGF in angiogenesis and vasculogenesis, and of eNOS in the regulation of vascular dilation and constriction, we hypothesized that these molecules are involved in the etiology of the honeymoon period.

VEGF, a potent mitogenic and angiogenic factor (36), is one of the factors responsible for the differentiation and proliferation of endothelial cells during embryogenesis (11), and both its proper expression and that of its receptors are necessary for normal vascular development (12).The deletion of the gene that encodes VEGFR-1 in rats results in vascular hyperplasia and failure to form functional vasculature, leading to death around GD 8.5. However, with the deletion of the kinase domain without affecting the extracellular domain, there is normal vascular development (37). Such findings suggest that VEGFR-1 functions as a decoy for VEGF, restricting its access to VEGFR-2 (37, 38). VEGFR-1 is also believed to play a role in the maturation and maintenance of vascular integrity (13), and its inhibition promotes VEGFR-2 expression (12). The deletion of the VEGFR-2 gene leads to early embryonic death due to a deficiency in vascular formation (14). There is a reciprocal regulation between VEGF and NO (15, 38–41). NO, synthesized by endothelial cells, is a critical factor in maintaining low vascular resistance in the pulmonary circulation (42, 43), which was demonstrated experimentally by Steudel et al., who recorded PH in mice with eNOS deficiency (44).

The publications that refer to VEGF expression, its receptors and eNOS in the CDH context present divergent results. As for VEGF, some studies have not observed changes (11, 16, 42, 45), although Boucherat et al. described very high levels in some neonates with CDH (42). Others found reduced (46, 47) or increased levels (7, 33, 48, 49). Few studies in the literature have evaluated the expression of VEGFR-1/2 in the context of the CDH. Previous studies carried out in our laboratory found decreased expression of VEGFR-1 (16, 27, 50). There are reports of increased VEGFR-2 in sheep (49) and reduced in mice (11, 50). Gallindo et al. studied this receptor's expression immediately after birth and after 30 min of ventilation and found reduced expression after ventilation in the groups with CDH (16).

The expression of eNOS in CDH is another point where the literature is controversial. While some studies have shown no difference (26, 43, 51), others found reduced (42, 49, 52–54) or increased expression in CDH (5). Regarding the effect of mechanical ventilation, Shinkai et al. found an increase in eNOS mRNA during the first hour of ventilation, followed by a decrease after 6 h (55). We hypothesized that the conflicting results found in the literature are related to the degree of PH presented and the ventilatory parameters used.

The increase of VEGF expression after 30 min of ventilation in rats with CDH could represent an attempt to “return to normality” (stimulating vasodilation), which cannot be sustained since there is a decrease in expression at 90 min.

As VEGFR-2 is the receptor that, despite the lower affinity for the ligand, presents more significant tyrosine kinase activity, the hypothesis is that its altered expression contributes to the vascular changes found in the CDH. Considering the IHC results, reduction of receptor expression in CDH after ventilation for 30 min corresponded to the decrease in MWT, and increase of the expression with the progress of the ventilatory time (60 and 90 min) corresponded to its increase, suggesting a central role of the VEGFR-2 in the pathogenesis of CDH-related PH.

There was a reduction (significant by IHC) of VEGFR-1 expression in the groups with CDH compared to controls. Because VEGFR-1 is believed to act as a decoy (37, 56), the already elevated VEGF would be more available for binding with VEGFR-2, promoting vascular proliferation stimulation (33), which could be one of the triggers for the end of the honeymoon period. Moreover, eNOS does not have its activity increased in the rat pups with CDH, which was expected to happen by VEGFR-2 stimulation.

In summary, we show an increased VEGF expression in CDH after 30 and 60 min of mechanical ventilation associated with decreased VEGFR-1 expression. We hypothesize that the reduced VEGFR-1 could leave the more free VEGF to ligate to VEGFR-2 leading to increased eNOS expression and vasodilation. However, in rat pups with CDH, there was an increase in the MWT (vasoconstriction), showing maladaptation to extrauterine circulation.

These results support the theory of a disruption of VEGF-NO expression and signaling in newborns with CDH (5, 11, 49, 57), which could be lead to altered vascular reactivity, suggesting a critical VEGF-NO role in the honeymoon period of experimental CDH.

This is the first study to evaluate VEGF, VEGFR-1/2 and eNOS during the honeymoon period in experimental CDH. Further studies are necessary to elucidate why newborns with CDH did not have increased eNOS despite increased VEGFR-2 expression. Understanding the disruption in this VEGF-NO pathway could provide new insights and a potential target for new treatment strategies.

The study has some limitations: (1) it is possible that we did not find statistical differences in WB due to the sample size. However, the number of neonates was determined mainly by the difficulty in ventilating patients with CDH for 60 and 90 min; (2) we did not expand the lungs for evaluation because there is no difference in the histological assessment for hypoplastic lungs in the rats' toxicological model (58), and (3) gene expression was not studied; however, not always it correlates with protein expression.

## Conclusion

Understanding the pathways that trigger the PH in CDH is one of the critical points for improving the treatment of newborns with CDH. Our findings show a change in the expression of VEGF, VEGFR-1/2 and eNOS in CDH induced by nitrofen after 30, 60, and 90 min of mechanical ventilation, suggesting that the VEGF-NO pathway plays an important role in the honeymoon period in experimental CDH and could be a target for novel therapies.

## Data Availability Statement

The raw data supporting the conclusions of this article will be made available by the authors, without undue reservation.

## Ethics Statement

The animal study was reviewed and approved by the Committee on Ethics in the Use of Animals of Ribeirão Preto Medical School (CEUA #088/2017).

## Author Contributions

KM and RF: conception and design of the study, acquisition of data, analysis and interpretation of data, and drafting the article. AF and LS: conception and design of the study, analysis and interpretation of data, and revising it critically for important intellectual content. CB, AS, and RR: analysis and interpretation of data, and revising it critically for important intellectual content. All authors contributed to the article and approved the submitted version.

## Conflict of Interest

The authors declare that the research was conducted in the absence of any commercial or financial relationships that could be construed as a potential conflict of interest.
